# Long-term Natural History and Elosulfase Alfa Treatment for Mucopolysaccharidosis Type IVA: A Single-Center Study in the Czech Republic

**DOI:** 10.21203/rs.3.rs-9023839/v1

**Published:** 2026-03-26

**Authors:** Robert Šáhó, Lenka Murgašová, Markéta Tesařová, Helena Poupětová, Igor Nestrašil, Jiří Zeman, Martin Magner

**Affiliations:** General University Hospital in Prague: Vseobecna Fakultni Nemocnice v Praze; University Hospital Kralovske Vinohrady: Fakultni Nemocnice Kralovske Vinohrady; General University Hospital in Prague: Vseobecna Fakultni Nemocnice v Praze; General University Hospital in Prague: Vseobecna Fakultni Nemocnice v Praze; University of Minnesota Department of Psychiatry & Behavioral Sciences: University of Minnesota Twin Cities Department of Psychiatry & Behavioral Sciences; General University Hospital in Prague: Vseobecna Fakultni Nemocnice v Praze; General University Hospital in Prague: Vseobecna Fakultni Nemocnice v Praze

**Keywords:** mucopolysaccharidosis type IVA, enzyme replacement therapy, natural history, long-term, spinal stenosis, cervical myelopathy

## Abstract

**Background:**

Mucopolysaccharidosis type IVA (MPS IVA, Morquio A syndrome) is a rare lysosomal storage disease primarily characterized by severe skeletal dysplasia. Clinical and laboratory data, including treatment responses, are reported for a cohort of Czech patients with MPS IVA.

**Results:**

Nineteen patients with MPS IVA from 17 families (13M/6F) were included; only one patient exhibited a mild form. The median age at disease onset was 3.0 ± 1.4 years, whereas the median age at confirmed diagnosis was 5.0 ± 3.5 years. At the time of diagnosis, all patients had skeletal symptoms (pectus carinatum in 58%, short stature in 47%, hip dysplasia in 47%), and 63% had nonskeletal symptoms. Each patient underwent at least one surgical procedure (77% knee, 59% spine, 41% adenotomy). On spinal MRI, 76% of patients presented with cervical stenosis, and 41% presented with cervical myelopathy. Eight patients received ERT for a median duration of 5.5 ± 2.6 years. Compared with non-ERT patients from natural history studies and non-ERT patients within our cohort, beneficial effects were evident in monitored parameters such as changes in the mean FVC and FEV1 volumes and the mean 6MWT distance. Additionally, the hidradenitis suppurativa observed in three patients improved with biologic therapy. We also describe two novel mutations (c.421_422dupTG, c.482G>C).

**Conclusions:**

Documenting the natural history of MPS IVA in this population may aid in early diagnosis. Despite the disease’s progressive nature, ERT was effective in our MPS IVA patients, demonstrating a consistent impact on endurance across all age groups.

## 1. Background

Mucopolysaccharidosis type IVA (MPS IVA), also known as Morquio syndrome type A (OMIM #253000), is a rare but significant lysosomal storage disorder inherited in an autosomal recessive manner. It is caused by mutations in the *GALNS* gene, resulting in a deficiency of the enzyme N-acetylglucosamine-6-sulfatase (GALNS; EC 3.1.6.4).[1] This enzyme deficiency results in the progressive accumulation of keratan sulfate (KS) and chondroitin-6-sulfate (CS) within lysosomes, particularly within connective tissues and the cornea,[2] ultimately driving the hallmark pathophysiology of the disease.

Pronounced skeletal abnormalities, including short stature, long-bone deformities, spinal curvature, pectus carinatum, and joint laxity, dominate the clinical presentation of MPS IVA.[1, 3] However, its manifestations extend beyond the skeletal system: patients often experience respiratory impairment, valvular heart disease leading to subsequent cardiac hypertrophy, corneal clouding, vision and hearing loss, and characteristic coarse facial features. [1, 4] The severity of these clinical features contributes to a highly variable life expectancy. Individuals with milder forms of MPS IVA may live 20–40 years, with some cases reported over 60 years. In contrast, severe phenotypes are often limited to late childhood or adolescence, with mortality typically resulting from spinal cord compression or respiratory failure.[5] The birth prevalence of MPS IVA is population specific, ranging from 1 in 140,000 in the Czech Republic to 1 in 872,000 in Australia.[6]

Since its approval in 2014, enzyme replacement therapy (ERT) with elosulfase alfa has been recommended as the first-line treatment according to international guidelines.[7] Clinical trials and registry data have shown that ERT improves quality of life, increases endurance, as evidenced by the 6-minute walk test (6MWT), supports respiratory function, enhances daily activity performance, and reduces urinary KS levels.[8, 9] Long-term stabilization of endurance and respiratory function has been reported in the Morquio A Registry Study (MARS).[10] However, ERT has not demonstrated efficacy in addressing the skeletal dysplasia and bone defects associated with MPS IVA.[11]

While natural history studies have documented the progression of MPS IVA, including a high rate of surgical intervention and a gradual decline in physical endurance,[2, 12–16] real-world data on the therapeutic impact of ERT remain limited.[17–21] This retrospective observational study reviewed the clinical history, genotypic characteristics, and phenotypic profiles of 19 Czech patients with MPS IVA since 1976. Additionally, we evaluated the effects of ERT in a subset of treated patients, providing valuable insights into the real-world outcomes of ERT in this patient cohort.

## 2. Patients and methods

Nineteen patients who were diagnosed with MPS IVA at the Department of Paediatrics and Inherited Metabolic Disorders, First Faculty of Medicine, Charles University and General University Hospital, Prague, Czech Republic, were included in this study. The diagnosis was confirmed through enzymatic and/or genetic testing. Patients in the cohort were observed longitudinally from 1976–2023, with data analysed at the conclusion of the study. Clinical, laboratory, and genetic data, including data from both outpatient and inpatient visits, were retrospectively collected from patient records.

### 2.1. Clinical Symptoms

Clinical data included the onset of the disease, initial presenting symptoms, age at diagnosis, age at the start of ERT, anthropometric measures, and the presence of hepatomegaly.

On the basis of MPS IVA-specific growth charts,[22] patients were assigned a “classic” designation to classify phenotypes if their height fell below the 75th percentile. Endurance was evaluated via the 6MWT following established guidelines.[23] Additional clinical evaluations included two-dimensional Doppler echocardiography, spinal MRI, audiometry, ocular examinations of the fundus and anterior segment, spirometry assessments for forced vital capacity (FVC) and forced expiratory volume in 1 second (FEV1), and polysomnography. The determination of urinary keratan sulfate quantity was unavailable due to laboratory limitations at our center.

### 2.2. Enzymology

White blood cell enzyme activity was measured in leukocytes isolated via dextran sedimentation and homogenized via ultrasonic disruption (Cole-Parmer CP-130, 30%) on ice. The protein concentration was determined via the Hartree method;[24]^,^[25] GALNS activity was assessed fluorometrically via the use of 4-methylumbelliferyl-β-D-galactopyranoside-6-sulfate (MU-β-Gal-6S) as the substrate.[26] β-Galactosidase activity was measured using 4-methylumbelliferyl-β-D-galactopyranoside (MU-β-Gal) as the substrate. MU fluorescence (Ex: 365 nm, Em: 448 nm) was detected on a luminiscence spectrometer (Perkin Elmer LS50B, Wellesley, U.S.A.).[27]

### 2.3. Molecular Genetic Analyses

Genomic DNA was extracted from blood samples, and all exons of the *GALNS* gene (NM_000512.5) were amplified and analysed by Sanger sequencing via the ABI3500xL Genetic Analyser (Applied Biosystems, USA).

### 2.4. Ethics

All data were accessed in compliance with relevant laws and ethical standards for the study period and in accordance with the Declaration of Helsinki, as revised in 2013.[28] Informed consent for genetic testing was obtained from all participants. Ethical approval for the study was granted by the Institutional Review Board (IRB) of the General University Hospital in Prague (Approval Number: 100/23 S-IV).

## 3. Results

This study involved 19 patients (13 males, 6 females) from 17 unrelated families, all with a confirmed diagnosis of MPS IVA. The cohort’s median age was 24.7 ± 13.0 years (range: 3.3–52.0 years), with a mean follow-up period of 18.9 ± 12.7 years (range: 1.3–47.1 years). Notably, patient 17 passed away at 30.5 years of age due to respiratory complications associated with COVID-19 infection. The median age at symptom onset was 3.0 ± 1.4 years (range: 4 months to 5.8 years), whereas the median age at confirmed diagnosis was 5.0 ± 3.5 years (range: 2–16.2 years), with a mean diagnostic delay of 3.1 ± 3.3 years (range: 0–11.2 years). For patient 19, an initial diagnosis of mucopolysaccharidosis was made at age 5 without subtype specification; the patient was later lost to follow-up and re-evaluated at age 48 when MPS IVA was confirmed.

Among the 19 patients, 8 (6 males, 2 females) received ERT with elosulfase alfa. The median age at the start of ERT was 6.0 ± 6.0 years (range 2.4–20.5 years). At the time of the study, the median duration of ERT was 5.5 ± 2.6 years (range: 11 months–8.9 years), representing a mean interval of 3.7 ± 5.0 years (range: 0.3–14.4 years) between diagnosis and the commencement of ERT.

### 3.1. Clinical outcomes

#### 3.1.1. Disease onset

**Figure 1:**
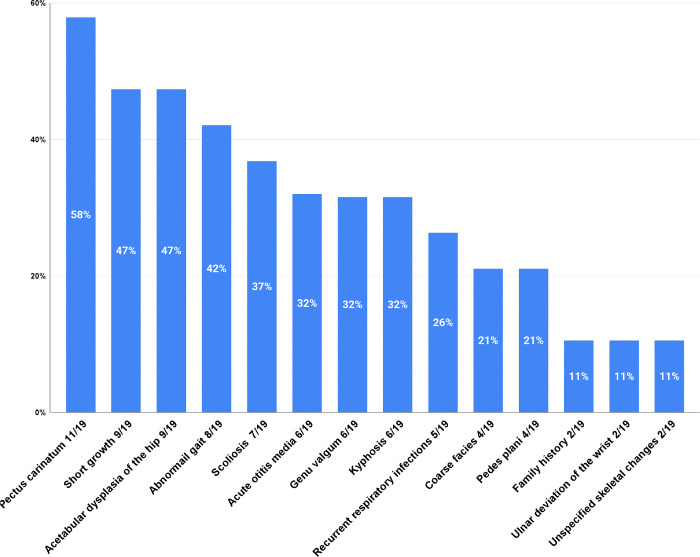
Initial symptoms in the cohort of 19 MPS IVA Czech patients

The most common initial symptoms observed in the cohort are depicted in Figure 1 and summarized in Table 1. Skeletal symptoms were universal at onset among all 19 patients, with specific findings including pectus carinatum (n=11), short stature (n=9), acetabular dysplasia of the hip (n=9), abnormal gait (n=8), scoliosis (n=7), genu valgum (n=6), and kyphosis (n=6). Additionally, 63% of patients presented initial nonskeletal symptoms, such as acute otitis media (n=6), frequent respiratory infections (n=5), and coarse facial features (n=4). In two patients, the diagnosis was prompted by a family history of MPS IVA. At the time of diagnosis, all patients with available radiographic data (n=17/19) presented classic features of multiplex dysostosis, whereas hepatomegaly was noted in only 19% of patients (3/16).

#### 3.1.2. Anthropometry and growth

Among the 18 MPS IVA patients whose birth data were recorded, the mean birth weight was 3,632 ± 534 g (range 2,650–4370 g), with an average gestational age of 38.8 ± 1.2 weeks. The average birth length in this group was 52.6 ± 3.0 cm (range 48–57 cm). On the basis of height assessments, 18 patients exhibited the classic MPS IVA phenotype, whereas one presented with an attenuated phenotype. Short stature was present in 85% (11/13) of patients at the time of diagnosis, with a mean height Z score of −3.2 ± 1.72 (range −5.34-−0.56). However, some patients initially grew within the normal percentile range (97th–25th percentile, data not shown) on Czech growth charts during the first 18 months of life. Patient 6, identified with an attenuated phenotype, had a Z score of 0.56 at the age of 5.5 years. At the last recorded visit, the mean Z score for height across the cohort (n=18) was −7.64 ± 2.87 (range −11.33 to −1.54), with patient 6 continuing to have a relatively high Z score of −1.54 (Table 1). When ERT-treated patients were compared with ERT-naïve patients (8 vs 11), the current mean height Z scores were −6.75 ± 3.47 and −8.35 ± 2.23, respectively, although this difference was not statistically significant (p=0.48). The baseline Z score for head circumference was 0.81 ± 1.18 (n=11).

#### 3.1.3. Endurance, spirometry, and assistive devices

**Figure 2:**
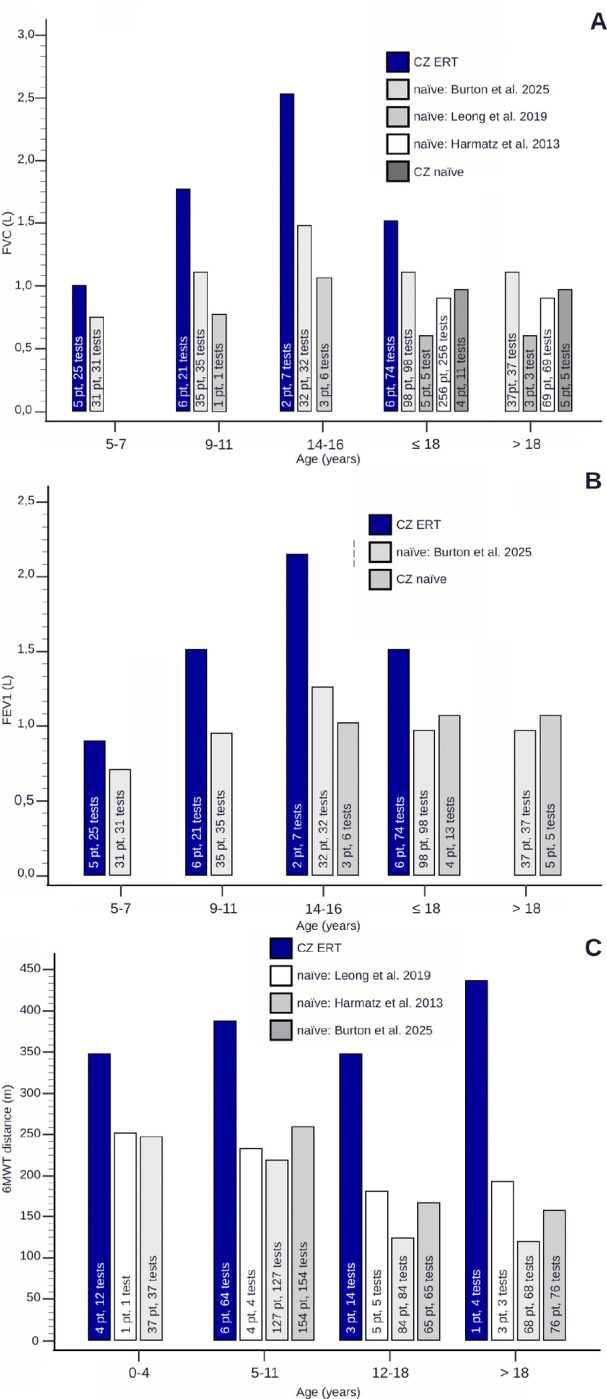
Spirometry and 6MWT results in the Czech MPS IVA cohort compared with published data (A- FVC, B- FEV 1, C- 6MWT)

Spirometric evaluations revealed higher mean values of FVC and FEV1 across all age groups in ERT-treated patients than in untreated patients in natural history studies [14,20,29] and in the untreated patients in our cohort (Table 2, Figure 2a, b). The differences in the FVC between the treated and untreated groups ranged from 1.05 L to 0.25 L, and the FEV1 ranged from 0.89 L to 0.18 L. Unlike the expected decline observed in natural history data, lung function remained stable or improved across age groups in our cohort, regardless of treatment status. Notably, direct statistical analysis was not possible due to the unavailability of source data from natural history studies.

#### 3.1.4. Mobility and Endurance Assessment

In the most recent follow-up, 10 patients (53%) were nonambulatory and relied on wheelchairs, with a mean age of 20.3 years when they ceased walking (data available for five patients). Additionally, three patients (16%) used a wheelchair for longer distances, whereas two (11%) required mobility aids such as walkers or crutches. Data for the 6MWT were not available for ERT-naïve patients in this cohort. However, ERT-treated patients demonstrated a greater mean 6MWT distance than did untreated individuals from natural history studies[14,20,29] across all age groups, with differences in distance between the treated and untreated groups ranging from 90.5 m to 315.7 m (Table 3, Figure 2c). Direct statistical analysis was not possible.

#### 3.1.5. Surgical history

All patients for whom data were available (n=17) underwent surgery at some point. The average age at first surgical intervention was 6 years. Among these patients, 13 (77%) required at least one knee surgery, whereas 10 (59%) underwent spinal cord surgery. Additional surgeries included adenotomy in 41% (7/17), hip surgery in 29% (5/17), and tympanostomy in 24% (4/17). Hernia repair and strabismus surgeries were performed in 6% of the patients each. The median ages at significant surgical intervention were as follows: knee surgery, 8.8 ± 6.1 years (5.5–35.5 years); spinal cord surgery, 14.0 ± 6.2 years (8.1–31 years); adenotomy, 3.7 ± 2 years (2.9–8.8 years); and hip surgery, 8.1 ± 1.4 years (6.4–9.7 years). Spinal magnetic resonance imaging (MRI) was available for 17 patients, revealing cervical stenosis in 13 (76%), cervical myelopathy in 7 (41%), and thoracic stenosis with odontoid hypoplasia in 5 patients (29%). Patient 4 experienced a significant decline in clinical status at age 15 following spinal cord surgery, which resulted in postoperative paraplegia and sensory impairment distal from the Th12 level. Subsequent complications included the development of a neurogenic bladder and a neurogenic bowel.

#### 3.1.6. Cardiologic evaluation

Echocardiographic data from the most recent follow-up were available for 18 patients. The most commonly observed cardiac anomalies were mitral and tricuspid regurgitation (both present in 56% of patients), followed by aortic regurgitation in 44%, aortic and tricuspid valve thickening in 50%, mitral valve thickening in 28%, and pulmonary regurgitation in 22%. The mean age of patients with valvular heart disease was 27.9 ± 11.3 years, whereas the mean age of those without valvular heart disease was 11.2 ± 4.7 years. Mild valvular disease was predominantly observed in patients aged 3.3−-18 years, whereas more advanced cases were observed in patients older than 18 years. One patient was identified with hypertrophic cardiomyopathy. The ejection fraction values remained within the normal range (≥55%) for all patients, with mean values of 71% at baseline and 68% at follow-up, indicating preserved systolic function.

#### 3.1.7. ENT Presentation, Vision, and Others

Among the 15 patients evaluated for ENT manifestations, 47% (7/15) experienced recurrent upper respiratory tract infections (RURIs), 20% (3/15) had recurrent otitis media (ROMA), and 47% (7/15) had at least one episode of acute otitis media (OMA). Audiometric assessments revealed hearing loss in 67% of the patients (10/15), with conductive hearing loss accounting for 53%, while the remaining cases were attributed to sensorineural and mixed hearing loss. Five patients were already using hearing aids, and the mean age at which hearing loss was documented was 12.4 years (n=8). Four patients had severe obstructive sleep apnea requiring nocturnal noninvasive ventilation (bilevel positive airway pressure (BiPAP)). Surgical interventions for ENT issues are summarized in the surgery history section.

Sixteen patients underwent a complete ophthalmologic assessment. Corneal clouding was observed in 75% of the cases, typically without vision impairment. No patients required corneal transplantation. One patient (6%) developed glaucoma. Cycloplegic refraction results revealed that hyperopia was the most common refractive error, affecting 69% of patients, followed by astigmatism at 63% and myopia, strabismus, and amblyopia, each affecting 19% of patients.

In addition, three patients had severe forms of hidradenitis suppurativa (HS) and were treated with biologic therapy. This condition is notably uncommon in lysosomal storage disorders.

### 3.2. Laboratory Data

#### 3.2.1. Enzymatic Analyses

All patients exhibited significantly deficient GALNS enzyme activity, with levels below 1% of the long-term control average (34,5 nmol/17 h/mg). In both the classic and attenuated phenotypes, GALNS activity remained below 1% of the control average (Table 1).

#### 3.2.2. Molecular–Genetic Analyses

Nineteen different variants of the *GALNS* gene were identified among 18 patients (12 males, 6 females) from 16 families. These variants included 13 missense mutations, two large deletions, two null variants (one small in-frame deletion/insertion and one intronic), and one duplication. The most common variants were c.1219A>C p.(Asn407His), which was found in 7 unrelated patients; c.1156C>T p.(Arg386Cys), which was present in 5 patients from 4 families; c.740G>A p.(Gly247Asp), which was found in 3 patients from 2 families; and c.860C>T p.(Ser287Leu), which was found in 3 patients from 2 families (Table 1).

Notably, Patient 17 presented with a rare codiagnosis of adenine phosphoribosyltransferase (APRT) deficiency, leading to 2,8-dihydroxyadenine urolithiasis, along with GALNS deficiency. Both the *GALNS* and *APRT* genes are located on chromosome 16q24.3, and genetic analysis revealed a deletion involving both genes. Polymerase chain reaction (PCR) confirmed that a novel junction formed by the fusion of sequences distal to GALNS exon 2 and proximal to APRT exon 3, with an estimated deletion size of approximately 100 kb, spanning *GALNS* intron 2 and *APRT* intron 2. This case was reported elsewhere.[30]

This study identified two unreported variants: a missense variant, c.482G>C (p.Gly161Ala), and a duplication, c.421_422dupTG. Notably, a different substitution at the same nucleotide position, c.482G>A, has been associated with an intermediate phenotype in a Chinese cohort.[31] With respect to duplication, a similar variant located one nucleotide upstream (closer to the intron) has been described previously.[32] Prediction algorithms provide conflicting interpretations of the impact of this duplication. Franklin and Varsome predict normal splicing with effects limited to amino acid substitution and a frameshift leading to p.Trp141Cysfs*13, suggesting that the impact may require reassessment. In contrast, SpliceAI predicts additional effects on splicing, indicating the potential for a more complex pathogenic mechanism.

Zygosity analysis revealed that 3 patients were homozygous, 1 was hemizygous, and 15 were compound heterozygous. Sixteen variants associated with the classic phenotype, whereas the variants c.740G>A p.(Gly247Asp) and c.1219A>C p.(Asn407His), were associated with both classic and attenuated phenotypes within this cohort.

Two novel variants, the Human Genome Mutation Database and ClinVar, have not been reported in public databases.

## 4. Discussion

This retrospective observational study provides a comprehensive overview of the clinical and laboratory characteristics and evaluates the effectiveness of ERT in a cohort of 19 Czech patients with MPS IVA. Most patients (18/19) exhibited the classic phenotype, with only one presenting an attenuated form. This represents a slightly higher prevalence than previously reported, which indicates the classic phenotype in approximately 75% of cases.[33] The age of onset in toddlerhood and the diagnostic delay observed in this cohort are consistent with other published MPS IVA data. Symptoms often emerge between 1 and 3 years of age, with the mean age of onset reported at approximately 2.0 to 2.6 years. Diagnostic delays are also similar to our findings of 4.7–6.9 years.[2,13,14]

As expected, skeletal symptoms, including pectus carinatum, short stature, acetabular dysplasia, and abnormal gait, were universally present. Although infants with MPS IVA are frequently born with average or above-average birth weight and length, growth failure often emerges in the second or third year of life.

Recurrent acute otitis media was a prevalent initial symptom in our cohort, underscoring that ENT symptoms, especially hearing loss, are relatively common in MPS IVA. In our cohort, 73% of patients experienced hearing loss, predominantly conductive hearing loss (two-thirds). Hearing loss in MPS IVA is reported in 67% to 94% of cases.[34–36] Consistent with other forms of MPS, younger patients predominantly exhibit conductive hearing loss, whereas sensorineural or mixed hearing loss becomes more prevalent with age.[37] A study by Riedner and Levin revealed that conductive hearing loss was universal in patients under 8 years of age, whereas older patients were more likely to develop sensorineural or mixed hearing loss.[36] Awareness of these ENT symptoms can facilitate earlier detection and long-term management by specialists, who play a crucial role in identifying hearing impairments potentially caused by ossicle deformity, recurrent upper respiratory infections, recurrent otitis media, and glycosaminoglycan accumulation. Although less often discussed, skin conditions such as hidradenitis suppurativa (HS) were observed in three patients (reported elsewhere[38]), although its exact pathophysiology in MPS IVA remains unknown.

Orthopedic and spinal surgeries pose significant challenges in managing MPS IVA. All patients underwent surgery, with a mean age of six years at their first procedure. Knee surgeries were the most common (77%), followed by spinal cord surgeries (59%). Cervical spine involvement, especially stenosis and instability, appears nearly universal in MPS IVA, often leading to myelopathy, paralysis, or sudden death. Previous studies reported varying overall surgery rates, ranging from 33% to 71%, underscoring an inconsistent approach to surgical indications. Broomfield et al. reported that 31% (26/82) of MPS IVA patients underwent cervical spine surgery at a median age of 6.1 years, emphasizing the importance of early intervention to preserve long-term neurological function.[39] The sparse literature lacks standardized recommendations for managing these orthopedic and neurosurgical challenges. The timing and indications for surgery are especially critical in spinal procedures, which have shown suboptimal outcomes, as observed in our cohort and other studies.[2] This clinical gap can be addressed through surveillance via spine MRI protocols sensitive to presymptomatic microstructural changes in the spinal cord, similar to those developed by the University of Minnesota group on the basis of their experience in degenerative cervical myelopathy.[40,41]

Genetic analysis revealed that missense mutations are the predominant type in the *GALNS* gene, which is consistent with findings from global databases. The c.1219A>C variant was the most prevalent variant in our cohort, accounting for 22% of all variants. This study also revealed two novel mutations (c.482G>C and c.421_422dupTG). The c.1156C>T variant, identified as the most common variant globally in the literature, was the second most frequent variant in our cohort.[42] Jezela Stanek et al. reported that heterozygotes for the c.121–9T>G variant exhibit less severe growth defects. [43] However, in our cohort, this was observed in only one patient with a mild disease form, whereas five others carrying the same mutation experienced severe growth impairment. This variant appears to be less commonly reported in the literature. In contrast, the c.860C>T variant, which was the fourth most common variant worldwide and the 3rd most common variant in our cohort, is notably the most prevalent variant among the Indian population.[42] Additionally, our findings suggest that combined APRT/GALNS deficiencies, although rare, may be more common than previously recognized, with two cases documented to date, including one in our cohort.[30,44]

The administration of elosulfase alfa to patients with MPS IVA in this study resulted in improved walking distances compared with data from age-matched naïve patients reported in the literature.[14,20,29] Data from several sources have also revealed the impact of ERT on the natural history outcomes of mobility issues in MPS, including the pivotal phase 3 randomized, double-blind, placebo-controlled MOR-004 clinical trial, which revealed statistically significant improvement in the 6MWT distance over placebo, which was sustained over 120 weeks.[8,45,46] In their review, Parini et al. reported improvement or stabilization of the 6MWT in MPS IVA patients after 1–3 years of treatment.[47] Pintos-Morel and colleagues reported improved walking distance in all but one cohort of patients after 8 months of ERT.[48] The mean 6MWT distance increased by 30% in four of six patients after ERT for 4.0–6.5 years in the study of Lin et al.[49] Other studies have also examined and reported improvements in walking distance and/or performance on the 3-minute stair climb test after the initiation of ERT.[50,51]

With respect to respiratory function, we focused on the characteristics of FVC and FEV1 across all age groups. We observed a general reduction in spirometry values in our patients compared with those in healthy individuals; however, the values remained stable or slightly improved under ERT, and the mean FVC and FEV1 values remained above those recorded in natural history studies.[14,20,29] Numerous studies have conclusively demonstrated that ERT appears to improve pulmonary function.[8,9,45,48,52] MARS, the longest and largest observational study of MPS IVA patients to date, also provided real-world evidence for long-term stabilization of endurance and respiratory function among ERT-treated patients.[10]

Mitral and tricuspid regurgitation were the most common symptoms of heart involvement in our cohort, with more advanced cases observed in patients older than 18 years. Only one patient manifested hypertrophic cardiomyopathy. None of our patients had been indicated for valve replacement yet. However, ERT likely has a negligible effect on cardiovascular outcomes, as no significant changes in heart structure or function have been noted.[18,53]

ERT had no effect on growth in our patient, whereas the youngest treated patient began therapy at the age of 2.4 years. Unfortunately, the effect of ERT on growth in MPS IV A has never been sufficiently documented.[51,54,55] We emphasize that the anthropometric data of our patients during the first 1.5−-2 years were normal. Therefore, normal growth at this age does not rule out the diagnosis of MPS IV A.

Our findings should be viewed in light of study limitations such as the limited number of patients, varying frequencies and intervals between clinical examinations, and incomplete data, particularly regarding functional tests from childhood in our adult patients. Furthermore, the absence of specific patient datasets from natural history studies precluded statistical comparisons. However, our study offers valuable real-world data on MPS IVA, which is essential for understanding treatment variability and optimizing clinical decisions.

## 5. Conclusions

Real-world data from Czech patients illustrate the effectiveness of enzyme replacement therapy in enhancing respiratory and endurance functions, although it has no observable impact on growth or skeletal structure. There is a pressing need for international guidelines concerning indications for skeletal and, in particular, spinal surgeries. Concurrent conditions, such as HS or APRT deficiency, are not uncommon in patients with MPS IVA.

## Figures and Tables

**Table 1:** Clinical, enzymatic, and molecular characteristics of the Czech MPS IVA cohort

Number / sex	Age at 1st symptom (y)	Age at dg.(y)	Current age (y)	ERT initiation age (y)	Initial symptoms		Height Z-score at dg.	Height current Z-score	GALNS activity (%+)	*GALNS* variants	
					Skeletal	Non- skeletal				1st allele	2nd allele
1/M	3	3.8	13	4.1	kyphosis, scoliosis	hepatomegaly, corneal clouding	−2.33	−5.2	0	c.151G>A (p.Glu51Lys)	c.1219A>C (p.Asn407His)
2/M	0.34	2.6	10	3.3	pectus carinatum, kyphosis, scoliosis, genu varum	coarse facies	−1.08	−4.68	0.2	c.421_422dupTG	c.1219A>C (p.Asn407His)
3/M	0.7	4.7	11.2	6.2	abnormal gait, pectus carinatum, acetabular dysplasia, kyphosis, scoliosis	recurrent acute otitis media, recurrent respiratory infections	−2.62	−4.81	0.5	c.502G>A (p.Gly168Arg)	c.1219A>C (p.Asn407His)
4/F	1.5	4.4	17.6	11.3	short growth, pedes plani, genu varum, pectus carinatum	-	−3.13	−10.6	0	c.1156C>T (p. Arg386Cys)	c.1156C>T (p. Arg386Cys)
5/M	2	5.2	11	5.7	pectus excavatum, pedes equinovarus, acetabular dysplasia, short growth	recurrent respiratory infections, recurrent acute otitis media	−3.28	−5.92	0	c.139G>A (p.Gly47Arg)	c.1520G>T (p.Cys507Phe)
6/M#	2	5.5	15.9	10.2	abnormal gait, - lower limb pain	-	0.56	−1.54	0.8	c.740G>A (p.Gly247Asp)	c.1219A>C (p.Asn407His)
7/F	3	6.1	22.3	20.5	short growth, pectus carinatum, genu varum, pedes plani, ulnar deviation of the wrist	-	−3.57	−11.33	0	c.482G>C (p.Gly161Ala)	c.1219A>C (p.Asn407His)
8/M	1.3	2	3.3	2.4	abnormal gait, pectus carinatum	coarse facies, delay of gross motor skills	−2,94	−9.94	0	c.1019G>A (p.Gly340Asp)	c.1483-15A>G (p.Asn495Phe
9/F	3.5	3.9	23.8	-	short growth, pectus carinatum	-	−4.84	−6.43	0	c.116A>G (p.Asp39Gly)	39 kb deletion ex1 part - ex 1
10/M*	5.8	5.8	24.7	-	abnormal gait, Skeletal changes without further specification	family history, recurrent respiratory infections	−5.34	−5.45	0.1	c.286G>T (p.Gly96Cys)	c.1156C>T (p. Arg386Cys)
11/M*	3	12.3	32	-	acetabular dysplasia, genu varum, short growth	-	NA	−9.09	0.3	c.740G>A (p.Gly247Asp)	c.860C>T (p.Ser287Leu)
12/M*	5	16.2	35.8	-	pectus carinatum, acetabular dysplasia, short growth	family history, recurrent respiratory infections	NA	−9.89	0.6	c.740G>A (p.Gly247Asp)	c.860C>T (p.Ser287Leu)
13/F	3	3	48.6	-	acetabular dysplasia, short growth	-	NA	−9.24	0	c.463G>A (p.Gly155Arg)	c.1219A>C (p.Asn407His)
14/F	3.5	3.9	32.3	-	pedes plani, genu varum, pectus carinatum	-	NA	−7.66	0.2	c.1156C>T (p. Arg386Cys)	c.1156C>T (p. Arg386Cys)
15/M*	1	9.1	27.9	-	Skeletal changes without further specification	coarse facies, recurrent acute otitis media	−5.27	−1,7	0.2	c.286G>T (p.Gly96Cys)	c.1156C>T (p. Arg386Cys)
16/M	3	5	36.3	-	short growth, kyphosis, pectus carinatum	coarse facies	NA	−4.56	0	c.1219A>C (p.Asn407His)	c.1219A>C (p.Asn407His)
17/M	NA	NA	† 30.5	-	acetabular dysplasia, pectus carinatum, kyphosis, scoliosis, genu varum, pedes plani	coarse facies	NA	−9.24	0	del (100 kb, GALNS in.2 to APRT in.	
18/M	3	5	26	-	short growth	recurrent respiratory infections	−4.31	−11.05	0	c.482G>C (p.Gly161Ala)	c.1156C>T (p. Arg386Cys)
19/M	3	5	52.1	-	acetabular dysplasia, pectus carinatum, kyphosis, scoliosis, ulnar deviation of the wrist	hernia, recurrent acute otitis media	−3.41	−10.84	0	c.853 855delTTC (p.Phe285del)	c.860C>T (p.Ser287Leu)

* Patients 10 and 15 are brothers, patients 11 and 12 are brothers

# only patient 6 had attenuated phenotype

+ residual activity in leukocytes, percentage oflong-term average of controls (34,5 nmol/17h/mg)

ERT – enzyme replacement therapy, F- female, GALNS - galactosamine (N-acetyl)-6-sulfatase M – male, NA – not available, , y – years

† death,

**Table 2:** Spirometry results in the Czech MPS IVA cohort compared with those of natural history studies*

Age range (years)	ERT-treated patients from our study		NATURAL HISTORY STUDIES				ERT-naïve patients from our study	
			Burton et al.^20^ ERT-naïve		Leong HY et al.^14^ ERT-naïve	Harmatz et al.^29^ ERT-naïve		
	FVC ± SD (L)	FEV1 ± SD (L)	FVC ± SD (L)	FEV1 ± SD (L)	FVC ± SD (L)	FVC ± SD (L)	FVC ± SD (L)	FEV1 ± SD (L)
5–7	1.0 ± 0.2 (25 tests, 5 pt)	0.9± 0.2 (25 tests, 5 pt)	0.75 ± 0.21 (n=31)	0.71 ± 0.19 (n=31)	-	-	-	-
9–11	1.77 ± 0.87 (21 tests, 6 pt)	1.51 ± 0.69 (21 tests, 6 pt)	1.11± 0.58 (n=35)	0.95 ± 0.45 (n=35)	-	-	0.77 (1 test, 1 pt)	-
14–16	2.53 ± 2.1 (7 tests, 2 pt)	2.15 ± 1.77 (7 tests, 2 pt)	1.48 ± 1.09 (n=32)	1.26 ± 0.92 (n=32)	-	-	1.05 ± 0.51 (6 tests, 3 pt)	1.02 ± 0.52 (6 tests, 3 pt)
≤ 18	1.52±1.07 (74 tests, 6 pt)	1.51 ± 0.92 (74 tests, 6 pt)	1.11 ± 0.62 (n=98)	0.97 ± 0.52 (n=98)	0.6 ± 0.1 (n=5)	1.1 ± 0.7 (n=256)	0.97 ± 0.38 (11 tests, 4 pt)	1.07 ± 0.67 (13 test, 4 pt)
> 18	-	-	1.49 ± 1.27 (n=37)	1.26 ± 1.03 (n=37)	0.9 ± 0.2 L (n=3)	1.5 ± 1.1 L (n=69)	1.59 ± 0.94 (5 test, 5 pt	1.42 ± 1.07 (5 tests, 5 pt)

* including 90 evaluations presented as volumes with means ± SD across age groups for ERT-treated and ERT-naïve patients relative to published natural history data. Forced vital capacity (FVC) and forced expiratory volume in the first second (FEV1) are shown in litres (L). Results are presented as means and standard deviations.

ERT- enzyme replacement therapy, pt - patients

**Table 3:** Results of the 6MWT in the Czech MPS IVA cohort compared with those of natural history studies *

Age range (years)	ERT-treated patients from our study	NATURAL HISTORY STUDIES		
		Leong HY et al.^14^ ERT-naïve	Harmatz et al.^29^ ERT-naïve	Burton et al.^20^ ERT-naïve
	6MWT average + SD (m)			
0–4	347.8 ± 98.5 (4 pt, 12 tests)	246.9 ± 87.4 (n=1)	251.6 ± 121.5 (n=37)	-
5–11	387.1 ± 94.7 (6 pt, 64 tests)	218.8 ± 98.2 (n=4)	232.5 ± 140.1 (n=127)	259.2 ± 102.1 (n=154)
12–18	347.8 ± 33 (3 pt, 14 tests)	123.7 ± 116.4 (n=5)	181.2 ± 177.3 (n=84)	167.2 ± 168.7^#^ (n=65)
> 18	436.3 ± 62.9 (1 pt, 4 exams)	119.3 ± 121.2 (n=3)	193.1 ± 148.5 (n=68)	157 ± 158.1 (n=76)

* displaying mean distances ± SD across different age groups for ERT-treated and ERT-naïve patients

# data from patients aged 14–16 years, 6MWT - 6-minute walking test , ERT – enzyme replacement therapy

## Data Availability

All data generated or analysed during this study are included in this published article.

## Abbreviations

6MWT: 6-minute walk test
APRT: Adenine phosphoribosyltransferase
BiPAP: Bilevel positive airway pressure
CS: Chondroitin-6-sulfateENT – Ear, nose, and throat
ERT: Enzyme replacement therapy
FEV1: Forced expiratory volume in 1 second
FVC: Forced vital capacity
GALNS: *N*-acetylglucosamine-6-sulfatase
HS: Hidradenitis suppurativa
KS: Keratan sulfate
MARS: Morquio A Registry Study
MRI: Magnetic resonance imaging
MPS: Mucopolysaccharidosis
MPS IVA: Mucopolysaccharidosis type IVA (Morquio A syndrome)
MU-β-Gal: 4-methylumbelliferyl-β-D-galactopyranoside
MU-β-Gal-6S: 4-methylumbelliferyl-β-D-galactopyranoside-6-sulfate
OMIM: Online Mendelian Inheritance in Man
PCR: Polymerase chain reaction
ROMA: Recurrent otitis media
RURI: Recurrent upper respiratory tract infections
